# Bridging the Orthopaedic Education Gap: Implementing a Fracture Conference Curriculum for Medical Students at Institutions Without Affiliated Residency Programs

**DOI:** 10.2106/JBJS.OA.26.00152

**Published:** 2026-09-22

**Authors:** Lachlan F. Anderson, Carl Berghult, Erica R. Olfson, Joseph Mellberg, Mitchel Hawley, Anne M. Boeckmann, David N. Shau

**Affiliations:** 1 The Anne Marion Burnett School of Medicine at TCU, Fort Worth, Texas; 2 Texas Hip and Knee Foundation, Fort Worth, Texas; 3 Department of Orthopaedic Surgery, University of Arizona College of Medicine, Tucson, Arizona; 4 Department of Orthopaedics, Emory University School of Medicine, Atlanta, Georgia

## Abstract

**Background:**

Access to orthopaedic education varies substantially across institutions, particularly at medical schools without affiliated residency programs. Faculty-supervised, peer-facilitated fracture conference curricula have emerged as a potential solution, yet evidence evaluating their impact remains limited. This study evaluated a structured, faculty-supervised fracture conference curriculum at medical schools without an affiliated orthopaedic department.

**Methods:**

A cross-sectional survey was administered to medical students who attended at least 1 session of a 13-module fracture conference curriculum between Fall 2022 and Spring 2026. The survey assessed pre- and postparticipation perceived familiarity across 7 orthopaedic skill domains, interest in orthopaedic surgery, agreement with 6 predefined statements evaluating educational value, and barriers to participation.

**Results:**

Forty-one respondents were included. Respondents reported consistent improvements in perceived familiarity across all 7 domains (all p < 0.001). Agreement with 6 predefined statements evaluating the program's educational value and impact had scores that were uniformly high, with respondents endorsing the low-stakes learning environment and the program's role in audition rotation preparedness. Among 10 respondents who completed an orthopaedic elective or away rotation, 90% rated the curriculum as “Very” or “Extremely” helpful.

**Conclusion:**

A structured, faculty-supervised fracture conference curriculum represents a potential approach to improving orthopaedic familiarity, specialty interest, and rotation preparedness among medical students at institutions without affiliated residency programs. This model may offer a low-resource, replicable framework for expanding orthopaedic exposure to students who currently lack access to specialty-specific education and warrants further evaluation through prospective, multi-institutional study.

## Introduction

Access to orthopaedic education during medical school demonstrates substantial variability across institutions and is frequently influenced by the presence of affiliated orthopaedic residency programs and structured departmental educational infrastructure^1-5^. Institutions lacking residency-affiliated orthopaedic departments, which includes 30 of the 157 allopathic programs, may offer limited access to formalized educational experiences that are generally found through established educational institutions^2,6^.

These structural limitations may disproportionately affect junior medical students, particularly at schools without an affiliated orthopaedic residency, given that nationally only 15% of medical schools require a mandatory orthopaedic rotation and 34% offer 1 as an elective^2,6-8^. At institutions with affiliated programs, early and structured exposure to orthopaedic surgery may be more readily available to students in their first and second years of training; students at nonaffiliated institutions, by contrast, often seek similar opportunities but lack consistent access to orthopaedic-specific learning environments^6,7,9^.

Beyond structural access, the culture of traditional orthopaedic teaching environments may itself present a barrier to early engagement. These settings may be perceived as intimidating by junior medical students, which can limit participation, question-asking, and opportunities for exploratory learning^10,11^. Learner-centered and psychologically safe educational settings may facilitate more active engagement and early specialty exploration, a consideration particularly relevant for students navigating unfamiliar clinical environments without the scaffolding of an affiliated department^10,12,13^.

Faculty-mentored, peer-facilitated educational models offer a potential solution by supplementing formal curricula and supporting preparation for clinical rotations. Prior studies have described similar initiatives directed at senior medical students in short-term formats of 1 to 3 months, but longitudinal programs directed at students across all training levels remain understudied^13-16^. This study, therefore, evaluated the outcomes of 1 such curriculum and its utility as a replicable framework for orthopaedic education at similarly situated institutions.

## Methods

### Study Design and Program Development

This was a cross-sectional, survey-based study evaluating medical student perceptions following participation in a structured orthopaedic fracture education program. The program was developed under the direct guidance of a fellowship-trained orthopaedic adult reconstruction surgeon, who served as faculty mentor and was responsible for all curricular content, including syllabus design and selection of fracture topics covered across sessions. Student leaders managed the operational aspects of the program, including scheduling, presentation formatting, and session facilitation. The program was established in Fall 2022 in a metropolitan area served by 2 medical schools without affiliated orthopaedic residency programs or departments and was open to students from any institution. Although the program was not formally affiliated with any medical school, a virtual attendance option expanded accessibility beyond the immediate metropolitan area, ultimately drawing students from 4 medical schools across the state, including both MD and DO programs.

### Setting and Participants

The program consisted of a 13-module fracture conference curriculum covering skeletal anatomy, fracture evaluation, radiographic interpretation, and applied orthopaedic principles, with the full syllabus detailed in the study by Boeckmann et al^13^. Curricular content was developed under faculty direction, informed by established orthopaedic educational resources including the OTA Fracture book^17^, AO Orthopaedics^18^, and free online resources such as Orthobullets. Radiographic examples were sourced from open-access databases with citations provided where relevant.

Sessions were held twice monthly, lasted approximately 75 minutes, and followed a structured active learning format. A fracture image was displayed, and students were given 60 seconds to discuss their interpretation with a partner before a randomly selected student presented their read and answered 3 follow-up questions. This was followed by a three-to-five minute focused lecture providing context and teaching points. The curriculum was delivered longitudinally and restarted annually, allowing incoming students to begin at the foundation of the sequence.

The faculty mentor is an employed surgeon within a subspecialty orthopaedic center that is part of a large hospital system without an affiliated medical school or residency program. An orthopaedic fellow associated with that center frequently attended sessions in a supervisory capacity. Orthopaedic residents from programs within the broader metropolitan area were occasionally present on a voluntary basis. All medical students who attended at least 1 session between Fall 2022 and Spring 2026 were eligible for survey participation. Students from all years of training were included, participation was voluntary, and no incentives were offered.

### Survey Development

A structured survey instrument was developed by the student leadership team and faculty mentor to assess perceived orthopaedic education access, self-reported rotation preparedness, and overall program satisfaction. Closed-ended items used 5-point Likert scales^19^. Open-ended questions invited free-text responses regarding educational value, psychological safety of the learning environment, and suggestions for improvement. The survey was piloted with a small subset of participants and refined before full administration. Only minor wording refinements were made following piloting, and all study participants completed the same final version of the survey instrument. See Appendix A for the full survey.

### Survey Administration

Surveys were administered electronically via a secure platform (Qualtrics, Qualtrics International Inc.). Reminders were sent at 1 and 2 weeks following initial distribution. Responses were anonymous.

### Statistical Analysis

Descriptive statistics were used to summarize demographic characteristics and Likert-scale responses. Pre- and postparticipation perceived-familiarity scores were compared using paired t-tests or Wilcoxon signed rank tests based on normality assessment. A p-value of <0.05 was considered statistically significant. All analyses were performed using R (Version 4.4.0, R Foundation for Statistical Computing).

## Ethical Approval

This study was reviewed and approved as exempt by the Institutional Review Board (IRB) at the investigators' institution (IRB#2026-71). All participants provided informed consent before completing the survey.

## Results

Forty-one of 61 (67%) medical students completed the survey. Attendees were from 4 medical schools, including both MD and DO programs. Respondent characteristics are summarized in Table I. Program engagement was high, with 90% of respondents participating for more than 6 months and 78% attending more than 10 sessions.

**TABLE I:** Participant Characteristics Including Year in Medical School and Duration of Fracture Conference Participation (*n* = 41)

	<3 Mo	3-6 Mo	6-12 Mo	>12 Mo
MS1	0	1	16	0
MS2	0	1	2	9
MS3	1	1	0	7
MS4	0	0	0	3

### Interest in Orthopaedic Surgery

Participation was associated with a statistically significant increase in self-reported interest in orthopaedic surgery. The mean interest scores rose from 4.15 ± 0.94 before to 4.63 ± 0.70 after participation (paired *t*-test: p < 0.001, *n* = 41).

### Reported Utility in Elective or Away Rotation Preparation

Among the 10 respondents who had completed an orthopaedic elective or away rotation, 6 had completed a local orthopaedic elective only and 4 had completed both a local elective and an away rotation. All were MS3 or MS4 students. Local orthopaedic electives were available; however, the medical school did not sponsor a home orthopaedic surgery residency program. Nine of 10 (90%) rated the fracture conferences as “Very” or “Extremely” helpful in preparing them for that experience, with 1 respondent rating them “Moderately” helpful.

## Program Attitudes and Perceived Value

**Fig. 1:**
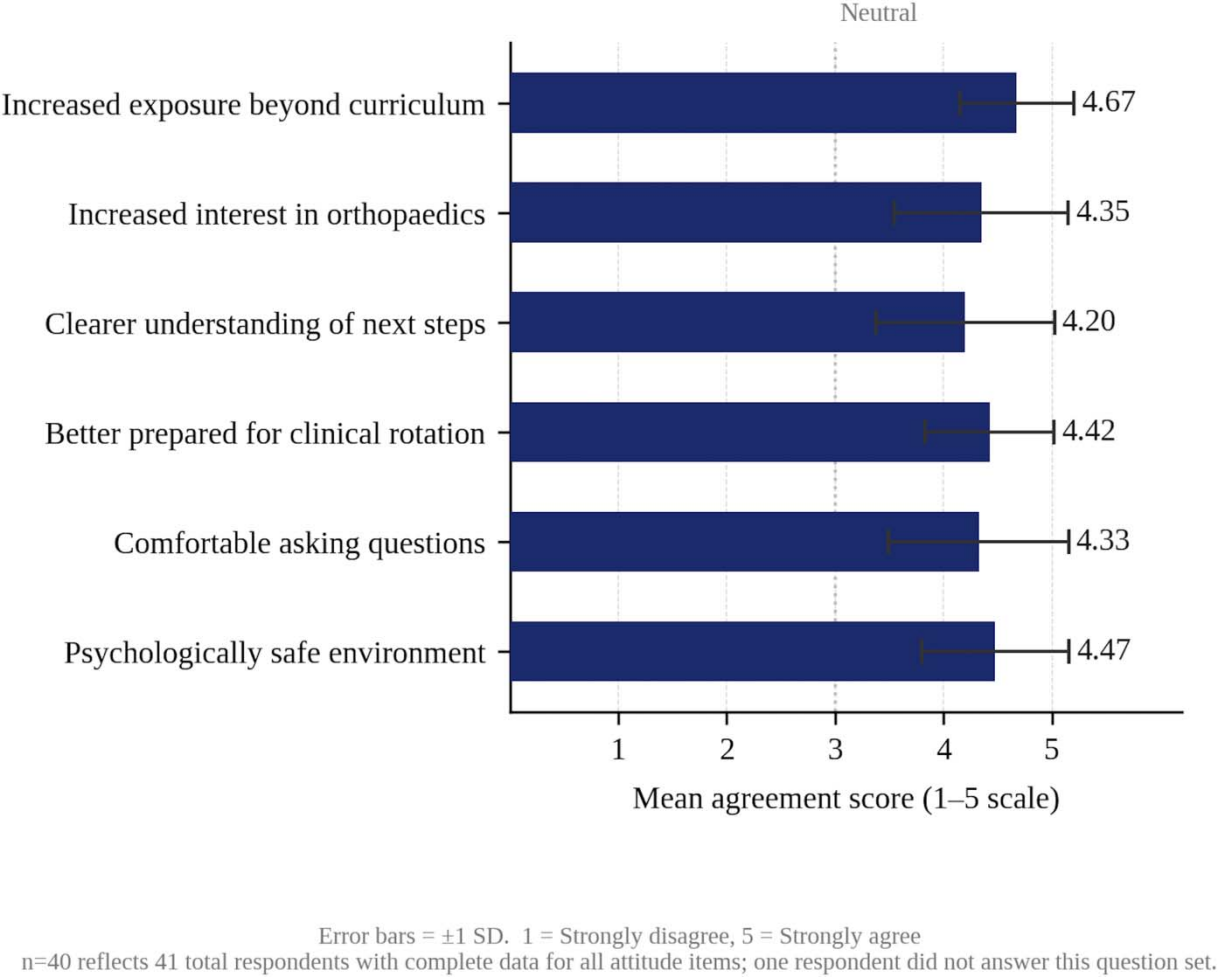
Agreement with program attitude statements following fracture conference participation.

Agreement with program attitude statements was consistently high across all 6 domains (Fig. 1). Respondents most strongly agreed that the conferences increased their orthopaedic exposure beyond the standard curriculum (mean: 4.67) and that they felt better prepared for an orthopaedic clinical rotation (4.42). Psychological safety was also highly rated (mean, 4.47), consistent with qualitative responses identifying the low-stakes learning environment as the most frequently cited valuable aspect of the program.

### Perceived Utility Across Orthopaedic Skill Domains

Respondents reported meaningful improvements in perceived familiarity across all 7 orthopaedic skill domains, with all paired comparisons reaching p < 0.001 (paired *t*-test, n = 41; Table II). Gains were largest in basic radiograph evaluation (Δ = +2.46) and fracture pattern identification (Δ = +2.24), while operative versus nonoperative management showed the smallest gain (Δ = +1.85) and lowest absolute postparticipation mean (3.32 ± 0.93), suggesting treatment decision making remains the most challenging domain at the medical student level.

**TABLE II:** Perceived Familiarity with Orthopaedic Skill Domains Before and After Fracture Conference Participation

Skill Domain	Before Mean ± SD	After Mean ± SD	Δ Mean	p
Radiograph evaluation	1.61 ± 0.77	4.07 ± 0.61	**+2.46**	<0.001
Fracture pattern identification	1.68 ± 0.91	3.93 ± 0.72	**+2.24**	<0.001
Operative vs. nonoperative management	1.46 ± 0.74	3.32 ± 0.93	**+1.85**	<0.001
Upper extremity anatomy	1.93 ± 0.96	3.93 ± 0.69	**+2.00**	<0.001
Lower extremity anatomy	1.95 ± 0.95	4.00 ± 0.63	**+2.05**	<0.001
Anatomy applied to clinical injury	1.77 ± 0.86	3.90 ± 0.74	**+2.12**	<0.001
Neurovascular structures at risk	1.71 ± 0.81	3.63 ± 0.86	**+1.93**	<0.001

Bold values indicate the mean change from preparticipation to postparticipation (postparticipation minus preparticipation).

### Barriers to Participation and Qualitative Findings

The most reported barriers to attendance were scheduling conflicts (n = 24, 59%) and time constraints (n = 14, 34%); content-related or participation hesitancy due to social apprehension were uncommon, with only 5 respondents (12%) reporting initial apprehension and only 2 (5%) citing content difficulty. In free-text responses, the most frequently reported valuable aspects were structured radiograph interpretation practice and the low pressure learning environment. When asked how the conferences influenced their decision to pursue orthopaedic surgery, 19 of 23 respondents (83%) who provided a free-text response reported a positive effect, with 3 noting the program had been a major or heavily influential factor in their career decision making.

## Discussion

Participation in a structured, faculty-supervised fracture conference curriculum was associated with meaningful improvements in perceived familiarity across orthopaedic skill domains, increased interest in orthopaedic surgery, and strong endorsement of the program's role in clinical rotation preparedness. These findings should be interpreted as preliminary and descriptive; this study was not designed to establish causality or demonstrate definitive educational efficacy but rather to characterize early participant perceptions of a novel program model. Barriers to participation were primarily logistical rather than educational or interpersonal, suggesting the model may be broadly accessible to the students who need it most.

Most participants were MS1 and MS2 students, a group that traditionally has limited clinical exposure to orthopaedic surgery, which is unsurprising, given the competing clerkship, residency application, and scheduling demands faced by MS3 and MS4 students^3^. For students at institutions without affiliated orthopaedic departments, these early learners may lack comparable opportunities for exposure, including departmental lecture series, resident-led teaching, or a structured clinical environment in which to observe the specialty. As such, this program may be particularly relevant for the student population with the greatest unmet need^3,5^. Notably, the curriculum also demonstrated early perceived value among upper-level students who completed an elective or away rotation, with 9 of 10 MS3/MS4 respondents (90%) rating it as “Very” or “Extremely” helpful in preparing them for that experience. This suggests that longitudinal preclinical exposure may enhance orthopaedic readiness across multiple stages of training, although larger prospective studies are needed to further evaluate this relationship.

A defining feature of this model, reflected in consistently high attitude scores (Figure 1), was the low-stakes learning environment conducive to active participation and question-asking. This is particularly meaningful in the context of orthopaedic education, where the well-documented hierarchical pressures and fear of public questioning in traditional teaching environments can limit engagement, particularly among junior students^20-22^. Unlike students at affiliated institutions who benefit from informal clinical exposure over time, those at unaffiliated schools may arrive on their first orthopaedic rotation having never discussed a fracture in a clinical context^21,23,24^. The peer-facilitated structure of this program, conducted under direct faculty supervision, attempts to create the conditions for underclassmen medical students to engage actively before that high-stakes clinical environment. Importantly, sessions were conducted under the direct supervision of an attending orthopaedic surgeon with supplemental supervision supplied by an orthopaedic surgery fellow and/or orthopaedic resident depending on availability, ensuring curricular accuracy and clinical relevance while preserving the accessibility of peer-facilitated delivery.

While self-reported perceived familiarity is not a validated measure of clinical competence, the consistent gains across all 7 domains serve to illustrate that participants found the curriculum broadly relevant and useful. The curriculum was intentionally designed around foundational concepts appropriate for the medical student level, including anatomy, fracture recognition, and radiographic interpretation, rather than advanced clinical decision making^25,26^. The relatively lower gains in operative versus nonoperative management, therefore, reflect appropriate curriculum design rather than a limitation of the model.

The primary contribution of this study is the description of this curriculum as a potentially replicable framework for orthopaedic education at institutions without affiliated residency programs. The model required no formal curricular integration and no institutional funding, sustained through a single fellowship-trained faculty mentor and a peer-facilitated student delivery structure, suggesting the barrier to implementation may be low. The modular design, twice-monthly cadence, faculty supervision, and student facilitation components are each independently adjustable to fit institutional constraints. The ability of this model to produce meaningful improvements in objective knowledge or downstream match outcomes remains to be established through larger, prospective, multi-institutional study.

## Limitations

There are several limitations. This was a single-site, cross-sectional study without a control group, limiting causal inference and generalizability. The survey relied on self-reported measures collected retrospectively, introducing recall and self-selection bias; notably, recall periods varied substantially, with early participants asked to recall baseline familiarity from up to 4 years prior. Because participation in the extracurricular conference was voluntary, the eligible survey population consisted of the 61 conference attendees. Accordingly, the survey response rate was calculated using these attendees as the denominator rather than the total medical school enrollment, which may limit the representativeness of the findings. This study did not collect data on downstream outcomes such as residency match, nor did it track whether reported increases in interest were sustained longitudinally among MS1 and MS2 respondents. Finally, the program's sustainability is heavily dependent on a committed faculty mentor willing to invest considerable uncompensated time in curricular oversight and session supervision across years of implementation; feasibility will vary based on institutional context and faculty availability.

## Future Directions

Future studies should incorporate prospective designs with objective knowledge assessments and longitudinal follow-up to determine whether gains in specialty interest translate into application and match outcomes. Multi-institutional replication would clarify generalizability, and hybrid or asynchronous formats warrant exploration given that scheduling conflicts were the most commonly cited barrier. This model may also have utility beyond its current target population, including fourth-year students preparing for internship or as a component of a structured orthopaedic boot camp curriculum.

## Conclusions

A structured, faculty-supervised fracture conference curriculum represents a potential approach to improving orthopaedic familiarity, specialty interest, and rotation preparedness among medical students at institutions without affiliated residency programs. This model may offer a low-resource, replicable framework for expanding orthopaedic exposure to students who currently lack access to specialty-specific education and warrants further evaluation through prospective, multi-institutional study.

## Appendix

Supporting material provided by the authors is posted with the online version of this article as a data supplement at jbjs.org (https://links.lww.com/JBJSOA/B353). This content was not copy-edited or verified by JBJS.
